# Toward a Digital Health Intervention for Vestibular Rehabilitation: Usability and Subjective Outcomes of a Novel Platform

**DOI:** 10.3389/fneur.2022.836796

**Published:** 2022-03-29

**Authors:** Dara Meldrum, Deirdre Murray, Roisin Vance, Sarah Coleman, Sonya McConnell, Orla Hardiman, Rory McConn Walsh

**Affiliations:** ^1^Academic Unit of Neurology, School of Medicine, Trinity College Dublin, Dublin, Ireland; ^2^Physiotherapy Department, Beaumont Hospital, Dublin, Ireland; ^3^Department of Neurology, Beaumont Hospital, Dublin, Ireland; ^4^Department of Otorhinolaryngology, Beaumont Hospital, Dublin, Ireland

**Keywords:** vestibular rehabilitation, digital health, exercise adherence, usability, enablement

## Abstract

Digital technologies are increasingly available and are reducing in cost. There is an opportunity to move to a digital health approach in vestibular rehabilitation (VR), but there is a paucity of suitable systems available and a consequent lack of evidence to support their use. This study aimed to investigate a novel digital platform developed specifically for VR (comprising clinician software, a wearable sensor, and a patient-facing app). Participants (*n* = 14, 9F:5M, mean age 59) with vestibular dysfunction and imbalance used the app for treatment, and therapists (*n* = 4) used the platform to deliver VR in the form of individualized exercise programmes over a mean of 17.4 ± 8.8 weeks. Outcomes included the system usability scale, the patient enablement instrument (PEI), change in subjective symptoms (numerical rating scales), percentage adherence to prescribed exercise, and a semi-structured interview on utility. A significant reduction was found in symptoms of vertigo/dizziness (*p* < 0.004), imbalance (*p* < 0.002), oscillopsia (*p* < 0.04), and anxiety (*p* < 0.02) after use. System usability scores were high for both clinicians (mean 85/100) and participants (mean 82.7/100) and high enablement was reported (mean PEI 6.5/12). Overall percentage adherence to the exercise prescription was highly variable and ranged from 4 to 78% when measured digitally. At semi-structured interviews, participants reported a high level of acceptance and satisfaction with digital delivery, and no adverse events were recorded. When COVID-19 restrictions eased, 2 participants trialed the head sensor with the application and found it highly usable. Further research is required to investigate the efficacy and how the wearable sensor impacts the delivery of care.

## Introduction

Across all fields in healthcare, digital health is on the rise, but its role in vestibular rehabilitation (VR) remains to be elucidated. The World Health Organization has defined digital health as “a broad umbrella term encompassing eHealth (which includes mHealth), as well as emerging areas, such as the use of advanced computing sciences in “big data,” genomics and artificial intelligence” (1). Within this definition, e-health is further defined as “the use of information and communications technology” and mHealth as “the use of mobile wireless technologies” (1). Traditionally, VR has been delivered face to face, with fewer than 5% reporting the use of telerehabilitation before the COVID-19 pandemic (2). One of the obvious benefits of digital health is to improve access, which is a challenge in VR (3). It is estimated that between 53 and 95 million adults have peripheral vestibular hypofunction across Europe and the US (4), and VR is a first-line, evidence-based treatment for these adults (5, 6). In a recent European Survey of VR in 22 countries, almost 50% of therapists reported that VR was difficult to access (2). Furthermore, the mainstay of VR is the home exercise programme. Gaze stabilization exercises are recommended at frequencies of 3–4 times per day (6), and meta-analysis of studies in fall prevention estimate a cut-off of at least 50 hours of targeted exercises to achieve therapeutic effects (7). It is likely not necessary and probably not feasible to have direct therapist supervision at these required intensities. Physical therapy fundamentally aims to improve movement and the ability to accurately track movement when the patient is exercising provides major opportunities for a better understanding of FITT (frequency, intensity, type, and time) principles. In turn, this would provide a much-needed evidence on the effectiveness of different components of exercise regimes. To advance the science of exercise prescription in VR, it is axiomatic that accurate data of exercise parameters are collected. This data would provide rich information for clinical and research purposes.

It is therefore anticipated that digital health will be embedded in future care, will solve the problems of access, and allow a greater understanding of exercise parameters. In a recent VR specific survey, 80% of therapists agreed that telehealth was an effective mode of delivering treatment but reported challenges with providing the written exercise programme and concerns about testing balance remotely with no caregiver present (8), and in a large survey of US physical therapists, 40% reported using telehealth software for the first time in 2020 (9). A benefit of the pandemic has been the requirement to use technology for daily human interactions beyond health care, and this has likely increased acceptance and familiarity with remote interactions. There are thus many opportunities for VR professionals to harness technology to solve the problems of prescribing and delivering exercise programmes remotely. However, therapists face many considerations when transitioning to digital care, and barriers are often cited as cost, IT infrastructure, data privacy and security, and uncertainty around efficacy (10, 11). Also, amongst therapists and patients, there remains a preference for face-to-face care, in a profession that is known for its “hands-on” approach (8).

Some studies are now appearing in the literature comparing conventional face-to-face VR with internet-based rehabilitation and have provided support for a digital approach and valuable insights on patient behavior and outcomes using digital technology. Geraghty et al. (12) and Pavlou et al. (13) both reported high attrition rates of 21 and 55%, respectively, in the groups allocated to unsupervised remote forms of VR, much higher than those who received face-to-face care. More recently, however, van Vugt et al. (14) reported similar attrition rates of ~5% in groups receiving internet-only based VR or therapist-supported home VR, perhaps heralding an increased acceptance of remote VR amongst patients.

Considerable challenges remain in proving efficacy and cost-effectiveness. The opportunity costs of a telerehabilitation infrastructure and associated technology (wearables, hardware, software, etc,) need to show a benefit for both the patient (in terms of time and money saved by being treated in the home, faster and better outcomes reducing loss of productivity, and increasing quality of life) and the health care provider (decreased consultation time, better outcomes, and more timely and targeted care). Therefore, it is not an easy task to integrate telerehab or technologies into rehabilitation. Major obstacles such as reimbursement by insurers also exist, although these are beginning to be addressed and will greatly assist with more widespread adoption.

The aims of this study were, first, to investigate the usability of a newly developed VR-specific digital platform and, second, to investigate its safety, exercise adherence, and outcomes with use.

## Materials and Methods

This was a usability study using a pre-treatment–post-treatment design to investigate the use of a novel digital VR platform for the rehabilitation of vestibular dysfunction and imbalance. The objectives of the study were to quantify patient and therapist usability of the platform, patient outcomes after using the platform, and to obtain, using a structured questionnaire, patients' views on the use of the app developed for the platform in their rehabilitation.

The study endpoints were:

1. The system usability scale (SUS) score (15) (patient and therapist) and the patient enablement instrument (PEI) score (16, 17) at 6 weeks (patient).
2. Change in visual analogue scores of subjective symptoms of dizziness/vertigo, imbalance, nausea, anxiety, and oscillopsia (18).
3. Percentage adherence to exercise (digitally measured by the application) and safety of the platform (number of adverse events).
4. Patient's views on the utility of the platform in their rehabilitation using a semi-structured phone interview (see Supplemental Material).

Data collection took place at the Neurotology Clinic and Physiotherapy Department at a large University teaching hospital. Ethical approval was obtained from the Hospital's Medical Research Ethics Committee. The study aimed to recruit 12–15 participants to gather sufficient data on patient's subjective views on using the platform. A sample size of 12 participants is deemed appropriate for usability studies (19).

Patients with vestibular dysfunction, confirmed with a positive video head impulse test (gain of <0.7 unilaterally) (https://www.synapsys.fr/en/home/), caloric testing (Canal Paresis > 20%), or in the absence of lab testing, a positive clinical head impulse test and nystagmus with fixation removed, and reporting at least one of the following subjective complaints: disequilibrium, gait instability, vertigo/dizziness, or motion sensitivity (20) were eligible for the study. They were excluded if they had previous VR, fluctuating vestibular disease (active Meniere's disease, migrainous vertigo), active benign paroxysmal positional vertigo, or other medical conditions in the acute phase (orthopedic injury). They were also excluded if they were unwilling or unable to use, or did not own a smartphone/tablet to use during the study.

Eligible patients were identified at the neurotology clinic by two of the researchers (RMW and DMu) who acted as gatekeepers and informed eligible patients about the study. Where there was a willingness to discuss the study, a third researcher (DMe) contacted the patient with information and obtained written informed consent after a cooling-off period of up to 1 week.

### Procedure

At baseline, participants underwent the following assessments. A physical in-person assessment was often not fully possible due to the COVID-19 second (October 2020) and third waves (January 2021), when some or all of rehabilitation moved to a telerehabilitation approach. Subjective assessments were collected remotely by the platform or during phone interviews and included the following:

1. The SUS (15) was designed as a subjective assessment of the usability of interface technologies. Levels of agreement with ten statements are scored using a five-point Likert scale anchored with “strongly disagree” and “strongly agree.” The SUS provides a point estimate of percentage usability. Scores above 70 are acceptable, and highly usable products score above 90. Scores below 50 indicate unacceptably low levels of usability. The validity, reliability, and sensitivity of the SUS have been extensively evaluated.
2. The PEI (16, 17) measures on a four-point Likert scale how enabled a patient feels to cope with their disease based on a consultation with their health care professional. Six questions are included and they enquire about the patient's understanding of illness, ability to cope with their illness and life, keep themselves healthy, and be confident about their health. A maximum score of 12 is attainable with higher scores indicating greater enablement. A score of 6–7 is considered to represent acceptable enablement (16, 17).

### Intervention

**Figure 1:**
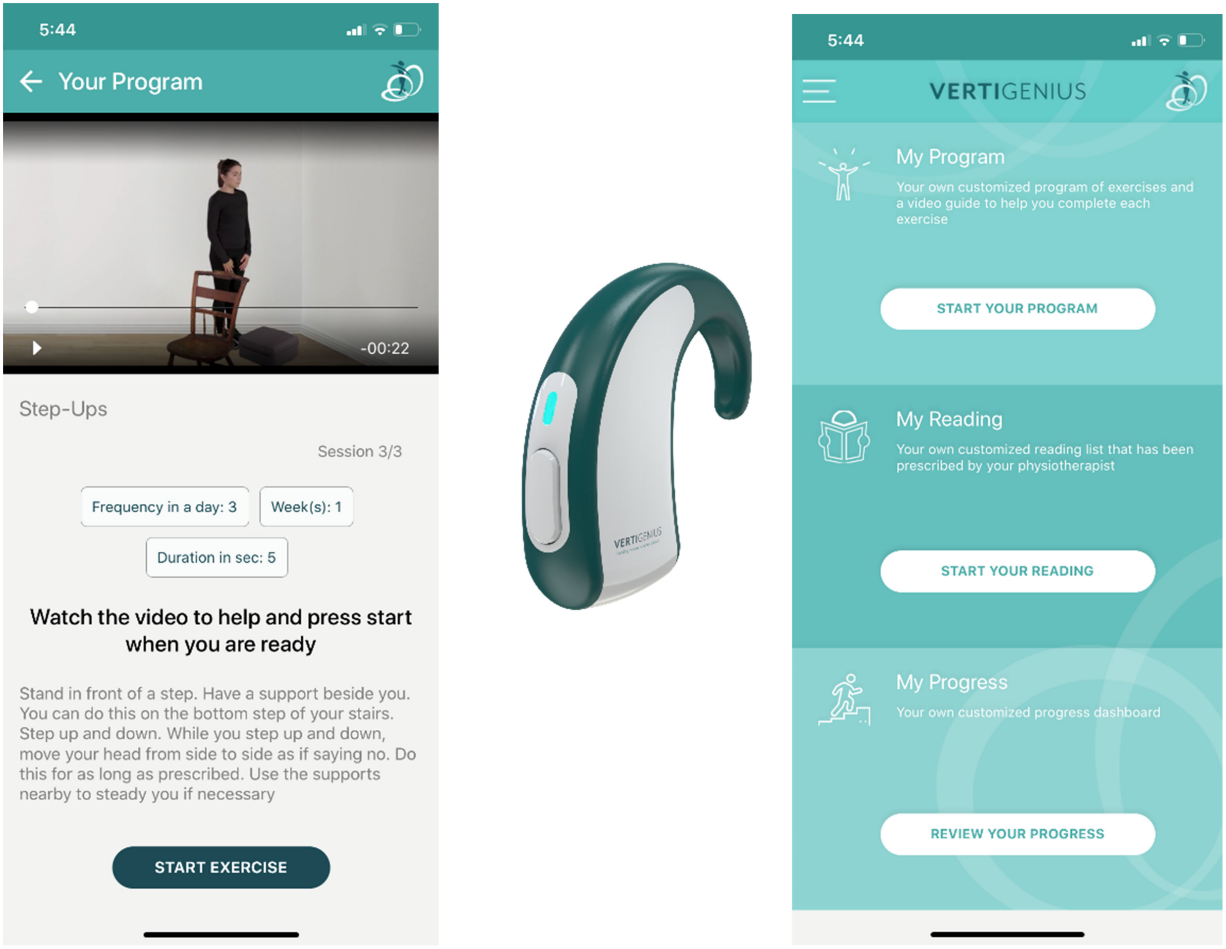
Patient-facing application and wearable head sensor.

Subsequent to completion of baseline measures and an initial assessment, the treating physiotherapist prescribed an individualized treatment programme on the platform which was then sent to the patient's smartphone and accessed *via* the App (Figure 1). The platform consisted of a digital clinical portal where the patient could be “on-boarded” and prescribed their individualized exercise programme. The exercises were broadly categorized into gaze stabilization, balance and gait, habituation, strengthening, breathing exercises (for anxiety), and optokinetic exercises. The FITT parameters could be individually adjusted by the therapist ensuring a customized individualized programme, which is considered the gold standard prescription approach (6, 21). Once prescribed, the patient application was used by the participant to perform their exercises; the app tracked the programme and symptoms, and these metrics were sent back to the portal in real-time. The participant was instructed on how to access and download the app to their smartphone or electronic tablet. Participants were pseudonymised on the clinical portal to prevent their identification by the technical personnel who were outside of the hospital setting. At each subsequent clinical visit (usually every week or 2 weeks) and until discharge, prescriptions were adjusted by the treating physiotherapist as appropriate. These visits mostly took place with a teleconsultation platform that was being used by the hospital during the pandemic.

The patient application on the smartphone provided the following information and support to the patient:

1. A video of each exercise with aural and text instructions.
2. Automatic guidance through their exercise programme. The app provided auditory feedback on head frequency during any gaze stabilization exercises (with an adjustable built aural metronome in the software).
3. Measurement of symptoms responses daily on a 10-point numerical rating scale (vertigo/dizziness, nausea and disequilibrium, anxiety, and oscillopsia). These were inputted by the participant once a day *via* the app. The first time a participant opened the app each day, the five scales would appear and they would be prompted on the screen to select the score that best described their symptoms that day.

Finally, the therapist could also select specific educational materials on the portal relating to balance and inner ear problems that were considered beneficial for their particular presentation and send them to the patient app. These included reading materials about what to expect from VR, symptoms of dizziness, vertigo and imbalance, and safety guidelines for exercising.

After using the application for 2–3 weeks, the participant was contacted by a researcher to explore their view on its utility and to collect the usability and enablement scores.

When COVID-19 restrictions were lifted, two participants attended the clinic and trialed the head sensor (Figure 1) with the app. The sensor (VG02; www.vertigenius.com) consists of an inertial measurement unit (IMU) to measure inertial motion of the head during gaze stabilization exercises. The IMU contains a gyroscope to measure the angular velocity of head movement (°/s) with yaw and pitch axis orientation. This angular velocity is used to estimate accurately the frequency in beats per minute (BPM) of the head rotation on either axis.

The participants were provided with the head sensor which connected *via* Bluetooth to the smartphone app. During gaze stabilization exercises, the sensor gave real-time feedback on the prescribed vs. actual head frequency during their exercises. A traffic light system was employed. For example, if the therapist had prescribed a head frequency of 120 beats per minute, the target displayed on the screen that the participant was fixating on turned red if the head was moving at a higher frequency, yellow if slower, and green if correct. Post-session, they were interviewed on their views of head sensor use during rehabilitation and asked to score the usability of the whole system on the SUS.

### Data Analysis

Data relating to the participant's interaction with the application was processed by one of the researchers (DM). Descriptive statistics were used for the analysis of SUS and PEI scores. Data were examined for normality using histograms and QQ plots. Paired *t*-tests and Mann–Whitney *U-*tests were used to investigate pre- and post-treatment NRS scores in normally and non-normally distributed outcomes respectively. Data from the semi-structured questionnaire were analyzed descriptively.

## Results

The study took place from August 2020 to August 2021. In total, 14 participants (9F:5M mean age 59 years, range 38–76 years) were recruited to the study. Baseline data and demographics are shown in Table 1. All participants had evidence of vestibular dysfunction and age-related abnormality in at least one balance test at baseline. Due to the fluctuating COVID-19 restrictions on face-to-face visits, testing was not uniform. Participants used the application for 17.4 ± 8.8 weeks and were prescribed 5.3 ± 3.6 programmes during this time. Three patients dropped out of the study, one reporting they did “not like technology” and preferred to use conventional methods of pen and paper for exercise prescription. Another dropped out before starting to use the application reporting that English was not her first language. One more participant dropped out after 1.5 months without giving a reason.

**Table 1:** Participant baseline clinical data.

**Participant**	**Gender**	**Age**	**Clinical/laboratory assessment**	**Baseline abnormality of balance**	**Length of time with symptoms**	**Duration of rehab in weeks**
1	F	58	Positive Right cHIT, Left Beating Horizontal Nystagmus with fixation removed	SOFEC Abnormal: 6, 10, 10 Secs	14 months	11
2	F	53	25% Left Canal Paresis, Caloric Testing	Condition 5 on SOT abnormal: Scores 0, 50, 50	4 Years	12
3	F	53	Abnormal Left vHIT Gain of 0.70	Condition 5 on SOT abnormal: Scores 59, 40, 64	2 years	20
4	F	45	28% Left Canal Paresis, Caloric Testing	SOFEC denoted Abnormal	1 year	2
5	M	76	Positive Left cHIT, Right Beating Horizontal Nystagmus with fixation removed	Condition 5 on SOT abnormal: Scores 0, 0, 26	1 year	30
6	M	38	32% Left Canal Paresis, Caloric Testing	Conditon 5 on SOT abnormal: Scores 50, 57, 76	9 months	34
7	M	72	Positive R cHIT, Right Beating Horizontal Nystagmus with fixation removed	SLS denoted abnormal	20 years	9
8	F	60	23% Right Canal Paresis, Caloric Testing	SOFEC abnormal: Scores 2, 2, 2 Secs	6 years	16
9	F	62	Clinical Diagnosis of UVL in Notes, Lab data not available	SLS abnormal: 10 Secs Eyes Open, 4 Secs Eyes Closed	10 years	^**^
10	F	66	Positive Right cHIT, Left Horizontal Nystagmus with fixation removed	SLS denoted abnormal	2 years	17
11	M	74	Positive Right cHIT, Left Beating Horizontal Nystagmus with fixation removed	SOFEC abnormal: 6 secs, SLS abnormal: 0 Secs	3 months	18
12	F	61	Abnormal Right vHIT Gain of 0.30	Condition 5 on SOT abnormal: scores 0, 0, 56	4 months	21
13	M	38	Abnormal Left vHIT gain of 0.77, Positive Right cHIT	SLS abnormal: 4 Secs Eyes Closed	1 year	19^*^
14	F	73	Positive Right cHIT, Left Beating Horizontal Nystagmus with fixation removed	SOFEC abnormal 9, 11, 15 Secs	9 months	^**^

*cHIT, clinical head impulse test; vHIT, video head impulse test; SOFEC, Stand on Foam Eyes Closed; Secs, seconds; SOT, Sensory Organization Test (Equitest); SLS, Single Leg Stance Test*.

* *Dropped out without giving a reason,*

** *Dropped out of study*.

**Figure 2:**
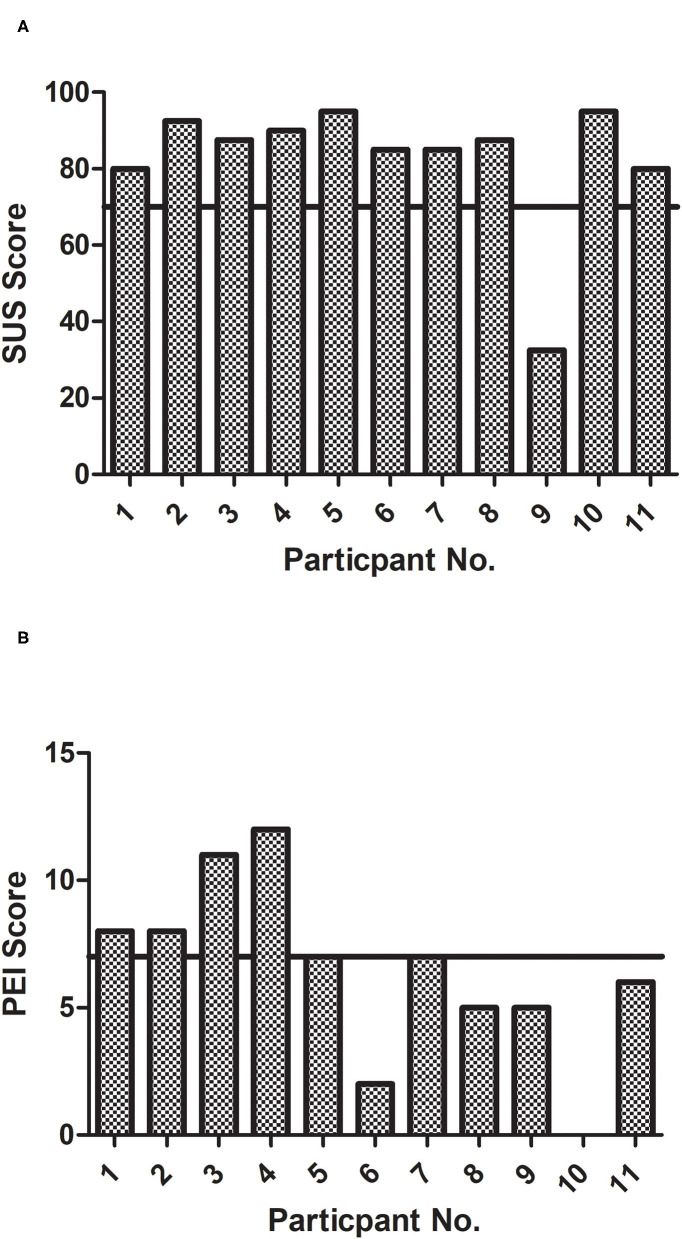
**(A)** System usability scale scores for each participant. The cut-off point of acceptable usability is shown on the y axis at 70/100 and **(B)** patient enablement instrument scores for each participant. The acceptable cut-off score of 7 is depicted by the line on the y axis at 7/12.

The average SUS was 82.7 ± 17 (range 32.5–92.5) (Figure 2A). Only one participant scored below 80. This was a female participant who scored 32.5 and who had dropped out due to a preference for conventional care.

### Change in Numerical Rating Scale Scores

**Figure 3:**
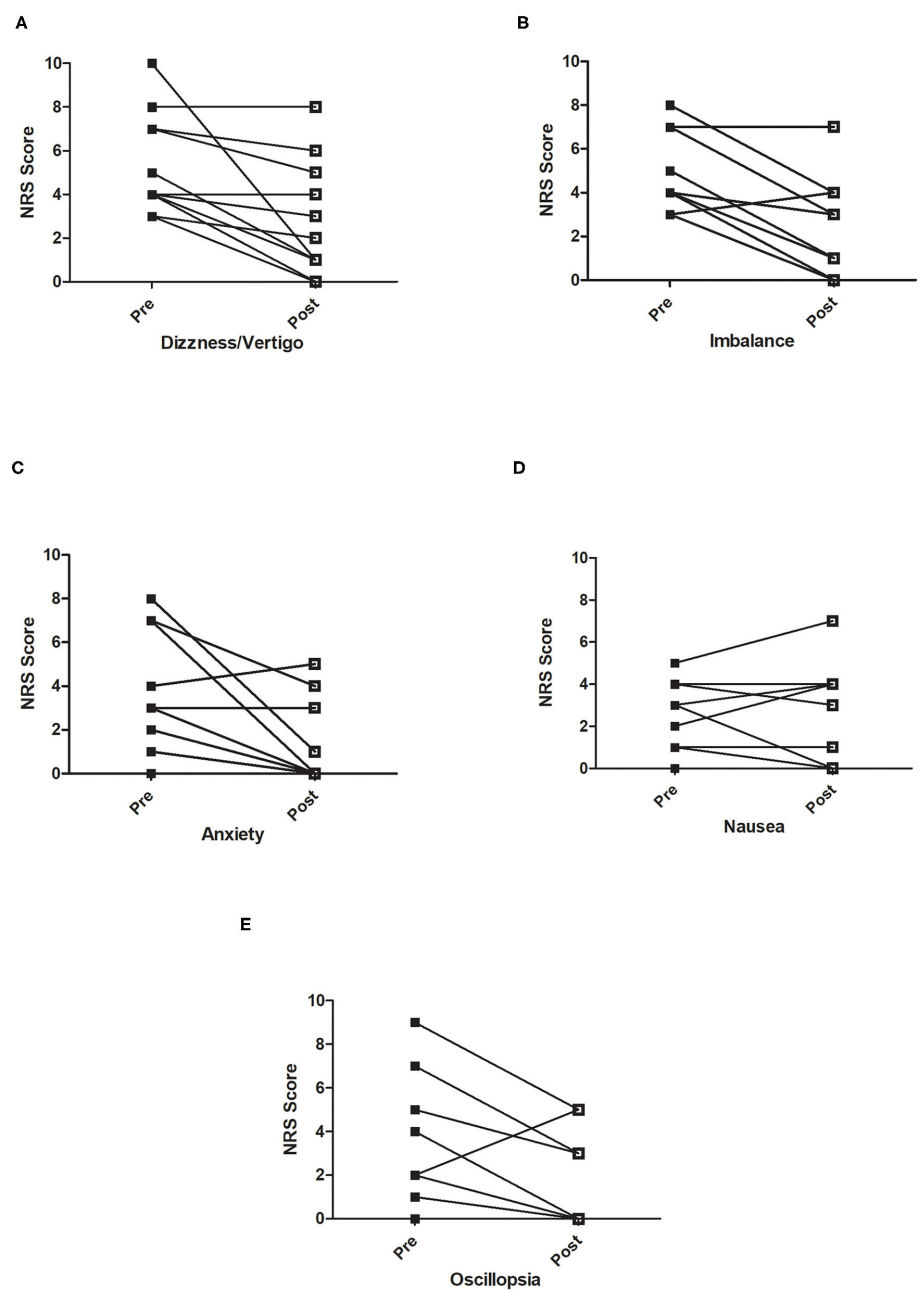
Pre- and post-treatment numerical rating scale scores for **(A)** Dizziness/vertigo, **(B)** Imbalance, **(C)** Anxiety, **(D)** Nausea, and **(E)** Oscillopsia. Participants rated symptoms daily on a 0–10 scale with 0 anchored as “none” and 10 as “symptom as bad as it could be.” Each participant is represented on a graph with closed squares showing the pre-treatment score and a line linking to the post-treatment score (open square).

The numerical rating scales were completed for the five subjective symptoms, namely, dizziness/vertigo, imbalance, nausea, anxiety, and oscillopsia. Figure 3 shows the pre–post treatment scores, and Table 2 shows the results of the paired *t*-tests for each symptom. All symptoms were significantly reduced by at least 40% (*p* < 0.05) (indicating improvement) except for the nausea NRS, which was reduced by only 9.1% (*p* = 1.0). No adverse events were recorded during the study.

**Table 2:** Mean change in numerical rating scale scores of subjective symptoms, where 0 is “no symptoms” and 10 is marked as “symptoms as bad as they can be.”

**Symptom**	**Pre-treatment** **NRS (/10) Mean (SD)**	**Post treatment** **NRS (/10) Mean (SD)**	**Change NRS (/10)**	***P*** **-Value**	**95% CI**
Vertigo/dizziness	5.26 (2.2)	2.6 (2.6)	2.67	0.004	1.1–4.3
Imbalance	5 (1.8)	2.8 (2.5)	2.25	0.002	1.0–3.4
Nausea	1.9 (1.8)	1.9 (2.4)	0	1.0	−0.9–0.9
Anxiety	3.2 (2.8)	1.1 (1.8)	2.2	0.02	0.4–3.7
Oscillopsia	3.1 (3.2)	1.6 (2.1)	1.5	0.04	0.1–2.9

### PEI Scores

The mean PEI score was 6.5 ± 3.7. One participant scored 0/12, which is a very low score indicating little or no enablement with treatment. On closer inspection of their numerical rating scores, improvements were seen in all five except the complaint of imbalance, which did not change. Another participant scored 2/12 but all numerical rating scores were improved. The remainder had acceptable PEIs (Figure 2B).

### SUS Scores: Health Care Practitioners

Four physical therapists used the platform during the study. Each completed a SUS. The mean score was 85 ± 10.2 (range 70–92.5). Two specifically reported needing time to become familiar with the application to onboard the patients, but after that phase they found it highly usable.

### Adherence

Once an exercise was completed, i.e., the patient opened and completed each exercise on the app, data on completion was sent back to the portal immediately allowing the therapists to easily view adherence in a graphical form. The average percentage adherence in counts of prescribed exercises (the frequency per day of each exercise multiplied by the duration of the programme in weeks) was 30.3 ± 23.5% (range 4.4–77.8%).

### Results From Semi-structure Interviews

Eleven participants completed a phone interview about using the application for their phone. Ten (91%) reported they would recommend the app to others for rehabilitation (reporting “yes definitely,” “yes absolutely,” and “yes 100%,” amongst other responses). One participant had macular degeneration and visual impairment and was using the app on an iPad. She specifically reported she liked accessing exercises on the iPad as “they were there at a touch, didn't have to fumble around with paper and if it was on paper, I wouldn't have been able to see it.”

A female participant who dropped out before using the app reported she “maybe” would recommend it. This participant was “afraid” to log in to the application, had needed help from a family member to download the app, and reported being “just lost” when trying to log in. This participant reported not “being one for apps” and not “a phone person.” A lower PEI score (5/12) and a lower SUS score (32.5) were recorded by this participant

Broad themes relating to the question of what participants liked and disliked about the app are shown below in Table 3.

**Table 3:** Results from the semi-structured interviews on the utility of the app in vestibular rehabilitation.

**Likes**
**• High ease of use, even for those who self-declared they were not “big” app users and had not used them for treatment before**.
**• High perceived usefulness of videos to correct performance, ease of access to the treatment (a few reporting its in “your pocket,” easier to follow than a “sheet of paper.”**
**• Benefits of the app over pen and paper adding structure and motivation to the exercise program. Comments on the “age we live in, it's all about the phone.”**
**• High levels of satisfaction with color scheme, simplicity and clarity of layout- app was easy to navigate**.
**• Improved self-efficacy in exercise, reports that the app was tangible, accessible in the home, reduced the anxiety of not performing exercises correctly, helped them remember to do exercises, stay “on-top” of the program**.
**• Educational materials were useful**.
**• Reduced the need for clinic visits (COVID-19 related concern)**.
**• Information on progress in the app and symptom collection allowed me “to see some days are better than others” and “liked that it was telling me I was making progress.”**
**Dislikes**
**• Occasionally technical problems (screen freezing)**.
**• There was no audible prompt for timer when doing balance exercises with eyes closed**.
**• Sometimes it was not clear that they had completed exercises**.
**• Unable to use the app and have others working in the background (for example listening to music when doing their walking program)**.
**• Symptoms only being recorded once a day was an issue for *n* = 2 who pointed out the exercises often increase the symptoms and they would like this to be recorded**.
**• No specific information/feedback provided by the app as to whether they were performing the exercise correctly**.
**• Requests to see more information on progress and mindfulness or other strategies for anxiety**.

### Head Sensor Usability

For the two participants who were observed using the head sensor during their prescribed exercise programme, SUS scores were 97.5 and 100, respectively, indicating very high usability. Both had been using the app only with the inbuilt metronome at home, and both remarked that the head sensor made the system “much better.” One participant likened the head sensor to a hearing aid and remarked it might be easily lost due to its small size. It was also observed that instructions were needed for participants to place the head sensor correctly on the ear. Participants reported that the visual feedback correcting their head frequency in real-time was helpful and the traffic light visual feedback was easily understood. They also stated that the head sensor could be easily incorporated into treatment at home and that they were in favor of the feedback on their performance and the additional information on their progress that could be obtained from the head sensor.

## Discussion

This study was performed to investigate both clinician and patient usability and experiences when using a novel digital health platform for the delivery of VR. During data collection, Ireland experienced waves 2 and 3 of the COVID-19 pandemic, which resulted in challenges to the recruitment process (recruitment is usually done face to face with baseline outcome measures performed in clinic). However, the platform easily enabled remote treatment in the home, and whether the participant had face-to-face or remote sessions with their therapist, did not negatively impact its use. In agreement with others, the study found that the benefits of digital delivery were augmented during COVID-19, conferring a feeling of safety and continuation of care when face-to-face visits were curtailed (11, 22). In addition, positive outcomes on the distressing subjective complaints of dizziness/vertigo, oscillopsia, imbalance, and anxiety were evident.

For “naïve” patient users, the app had high system usability scores. Generally, a score above 70 is deemed acceptable, and the mean score in this study was 83 (19, 23). There were three drop-outs, one did not speak English as a first language and one declined to use the application after downloading it as she was “just lost.” This was expected as technology is difficult for some.

High levels of enablement were also recorded by participants. Enablement is a key feature for patients to feel they can cope with and understand their illness. Scores of 6–7 are considered acceptable and the mean score was 6.5. Scores were higher than reported in other studies who reported lower mean scores of between 3 and 4 (24, 25).

All participants, except one, reported they would recommend the app to other patients with dizziness and vertigo. A high level of user acceptance was evident, and participants had little fault to find with the ease of use, layout, and look and feel of the application. The app can be considered to be usable.

For the 4 “naïve” Health Care Practitioners, the clinician portal also had high SUS scores at 83.3. They reported that it was easy, intuitive, and quick to use, and it streamlined and facilitated care remotely, the latter being a key benefit during the pandemic.

There were no adverse events recorded during the study. Participants used the application for an average of 17 ± 8.8 weeks, and there was preliminary evidence that a reduction in subjective symptoms of dizziness/vertigo, imbalance, nausea, anxiety, and oscillopsia occurred with the usage of the application, indicating the effectiveness of the treatment, in line with what is expected with VR. Due to COVID-19, we were unable to systematically collect physical outcome measures, and this must be evaluated in future studies to determine efficacy.

Two participants were non-English speakers, one of these dropped out due to difficulties with the English language and comprehension, but the other was able to engage with the patient app. It is thus a recommendation that when employing patient apps they are translated into the local language.

One key benefit of the platform was the automated collection of exercise adherence. Diaries are commonly used to this end but are frequently criticized as being inaccurate and time-consuming. This is the first study to report actual adherence to exercise and found a low mean adherence to exercise of 30%. This may be because the recommended frequency for gaze stabilization exercises in VR programmes is 3–4 times per day (6). However, significant improvements were seen in subjective symptoms, and future work is planned to investigate the minimum exercise adherence necessary to achieve benefits. Therapists personalized the exercise prescription according to participants' presenting impairments, and whilst this meant participants completed different exercises, this is the recommended practice (6, 21). However, all participants were prescribed gaze stabilization exercises, balance, and gait exercises. Further study of the effects of individual exercises with controls is required to determine optimal programmes.

Only two participants used the head sensor during the study and in a face to face setting. Although reported usability was high and the integration of the head sensor providing real-time corrective feedback was easily understood, these findings can only be considered preliminary.

## Study Limitations

The study took place during the second (October 2020) and third waves (January 2021) of the COVID-19 pandemic, therefore the physical outcome measures that were planned for the study were not feasible to collect as the face-to-face contact with the researchers, which would be usual in data collection, was not permitted. More complete data on physical outcomes of balance and gait would have provided a valuable addition to the findings. We were unable to provide all the participants with this head sensor, but initial trials with two participants were favorable, and a future study is underway to investigate the effect of the head sensor. Several participants who did not use the head sensor reported they would like more feedback on their exercise performance and personalized direction on what to do if exercises increased symptoms. The wearable sensor was designed to address this problem so it will be interesting to see how it impacts treatment.

Finally, the sample size was small but considered adequate for usability studies. Larger sample sizes will be required to demonstrate efficacy and cost-effectiveness.

In conclusion, this usability study has provided initial evidence that a novel digital platform incorporating a clinician portal and a patient-facing app are highly usable and accepted by health care practitioners and patients for VR. Significantly reduced symptoms were recorded with use, suggesting benefit. A “naïve” user of either can be easily onboarded and interact with the platform relevant to them. Importantly over the time frame of use, improvement in subjective symptoms was significant, supporting its use in vestibular and balance rehabilitation programmes. As with all applications, feature improvements were suggested, but the current workflows are usable and mirror the clinical pathway sufficiently to integrate the system easily into healthcare settings. Future studies planned include incorporating the platform's wearable sensor in a randomized controlled trial to investigate efficacy and cost-effectiveness.

## Data Availability Statement

The raw data supporting the conclusions of this article will be made available by the authors, without undue reservation.

## Ethics Statement

The study was reviewed by Beaumont Hospital Medical Research Ethics Committee, Beaumont Hospital, Beaumont Road, Dublin 9. The patients/participants provided their written informed consent to participate in this study.

## Author Contributions

DMe and DMu conceived the study. RM and OH were gatekeepers, recruited patients, and assisted with preparations for the Ethics Committee Submission. SM, SC, DMu, and RV treated the participants. DMe interviewed the patients and collected the outcomes, analyzed the data, and wrote the paper. DMe and RV commented on the manuscript. All authors contributed to the article and approved the submitted version.

## Funding

This work was supported by two Enterprise Ireland Research Commercialisation Funds (CF-2018-0966 and CF20201422-I).

## Conflict of Interest

DMe is the inventor of the digital intervention that was employed in the study. It is patent pending and she is named on the patent. This research is being commercialized and she is a shareholder in a start-up company formed before the end of the study. The remaining authors declare that the research was conducted in the absence of any commercial or financial relationships that could be construed as a potential conflict of interest.

## Publisher's Note

All claims expressed in this article are solely those of the authors and do not necessarily represent those of their affiliated organizations, or those of the publisher, the editors and the reviewers. Any product that may be evaluated in this article, or claim that may be made by its manufacturer, is not guaranteed or endorsed by the publisher.
