# Supplementary material for: Toward a Digital Health Intervention for Vestibular Rehabilitation: Usability and Subjective Outcomes of a Novel Platform
Source: Front Neurol. 2022 Mar 29;13:836796. doi: 10.3389/fneur.2022.836796 (PMC9001890; doi:10.3389/fneur.2022.836796)
Supplement: Supplementary file 1 [file Data_Sheet_1.PDF]

Vertigenius Patient Questionnaire

UIN________________________ Date____________________

1. How long have you been using the app?

2. Tell me about your experience to date the app?

3. What did you like?

4. What did you not like?

5. What did you find difficult about it?

6. What did you find easy about it?

7. How are you in general with apps on your phone? Do you use many?

8. What do you think about using the app for rehabilitation?

9. What aspects, if any did it help with for rehabilitation?

10. What would you add if you could?

11. Would you recommend it to other patients with dizziness and balance problems

    Yes Maybe No
